# Dietary Magnesium Intake Improves Insulin Resistance among Non-Diabetic Individuals with Metabolic Syndrome Participating in a Dietary Trial

**DOI:** 10.3390/nu5103910

**Published:** 2013-09-27

**Authors:** Jinsong Wang, Gioia Persuitte, Barbara C. Olendzki, Nicole M. Wedick, Zhiying Zhang, Philip A. Merriam, Hua Fang, James Carmody, Gin-Fei Olendzki, Yunsheng Ma

**Affiliations:** 1 Department of Preventive Medicine, Medical School of Yangzhou University, Yangzhou 225001, China; E-Mail: Jinsong.Wang@umassmed.edu; 2 Division of Preventive and Behavioral Medicine, Department of Medicine, University of Massachusetts Medical School, Worcester, MA 01655, USA; E-Mails: Barbara.Olendzki@umassmed.edu (B.C.O.); Nicole.Wedick@umassmed.edu (N.M.W.); jamie.zhang8@gmail.com (Z.Z); Philip.Merriam@umassmed.edu (P.A.M.); James.Carmody@umassmed.edu (J.C.); Effie.Chung@umassmed.edu (G.-F.O.); 3 Division of Biostatistics and Health Services Research, Department of Quantitative Health Science, University of Massachusetts Medical School, Worcester, MA 01655, USA; E-Mails: Gioia.Persuitte@umassmed.edu (G.P.); HuaJulia.Fang@umassmed.edu (H.F.)

**Keywords:** magnesium, insulin resistance, metabolic syndrome, epidemiology

## Abstract

Many cross-sectional studies show an inverse association between dietary magnesium and insulin resistance, but few longitudinal studies examine the ability to meet the Recommended Dietary Allowance (RDA) for magnesium intake through food and its effect on insulin resistance among participants with metabolic syndrome (MetS). The dietary intervention study examined this question in 234 individuals with MetS. Magnesium intake was assessed using 24-h dietary recalls at baseline, 6, and 12 months. Fasting glucose and insulin levels were collected at each time point; and insulin resistance was estimated by the homeostasis model assessment (HOMA-IR). The relation between magnesium intake and HOMA-IR was assessed using linear mixed models adjusted for covariates. Baseline magnesium intake was 287 ± 93 mg/day (mean ± standard deviation), and HOMA-IR, fasting glucose and fasting insulin were 3.7 ± 3.5, 99 ± 13 mg/dL, and 15 ± 13 μU/mL, respectively. At baseline, 6-, and 12-months, 23.5%, 30.4%, and 27.7% met the RDA for magnesium. After multivariate adjustment, magnesium intake was inversely associated with metabolic biomarkers of insulin resistance (*P* < 0.01). Further, the likelihood of elevated HOMA-IR (>3.6) over time was 71% lower [odds ratio (OR): 0.29; 95% confidence interval (CI): 0.12, 0.72] in participants in the highest quartile of magnesium intake than those in the lowest quartile. For individuals meeting the RDA for magnesium, the multivariate-adjusted OR for high HOMA-IR over time was 0.37 (95% CI: 0.18, 0.77). These findings indicate that dietary magnesium intake is inadequate among non-diabetic individuals with MetS and suggest that increasing dietary magnesium to meet the RDA has a protective effect on insulin resistance.

## 1. Introduction

Magnesium is a vital nutrient responsible for over 300 biochemical reactions in the body including regulation of blood sugar levels, blood pressure, and bone health [1]. Foods rich in magnesium are also typically high in dietary fiber such as dark green vegetables, legumes, nuts, and whole grains. A necessary cofactor in glucose metabolism, magnesium has been implicated in a number of health conditions, including metabolic syndrome [2]. In an updated meta-analysis of 13 prospective cohort studies involving 536,318 participants and 24,516 cases, an inverse association was significant between magnesium intake and risk of type 2 diabetes [pooled relative risk and 95% confidence interval (CI) = 0.78 (0.73–0.84)] [3]. Another meta-analysis of nine randomized double-blind controlled trials with a total of 370 patients with type 2 diabetes, who were given oral magnesium supplementation for 4–16 weeks, found that it may reduce plasma fasting glucose levels in the treatment groups compared with placebo [−0.56 mmol/L (95% CI, −1.10 to −0.01)] but had no effect on hemoglobin A1c between both groups [−0.31% (95% CI, −0.81 to 0.19)] [4]. Some cross-sectional studies and one longitudinal study showed an inverse correlation between magnesium intake and insulin resistance in diabetic patients and normal individuals [5,6,7,8,9]. Two clinical trials indicated that oral magnesium supplementation could improve insulin sensitivity or insulin resistance in non-diabetic subjects [10,11]. However, the majority of studies examined participants with diabetes, with few longitudinal studies exploring this relationship among non-diabetic individuals with metabolic syndrome (MetS).

A national study revealed that most U.S. adults failed to consume adequate intakes of magnesium in their diet [12]. Previous studies have yet to evaluate if insulin resistance is related to this failure in meeting the Recommended Dietary Allowances (RDA) for magnesium. The present study examined the longitudinal association of magnesium intake (from food) and insulin resistance in non-diabetic participants with MetS, while also exploring insulin resistance as it relates to the RDA guidelines for magnesium intake.

## 2. Methods

### 2.1. Study Sample

Details of the “Can Do” study methodology have been described elsewhere [13]. Briefly, participant recruitment commenced at the University of Massachusetts Medical School (UMMS), Worcester, Massachusetts, USA, in May 2009 and was completed in February 2013. The study protocol was approved by the UMMS Institutional Review Board for use of human subjects in medical research and all participants gave informed consent. A total of 234 non-diabetic men and women who had a BMI ≥30 and ≤40 kg/m^2^ and met three or more components of MetS [14], and did not have a fasting blood sugar >126 mg/dL, were enrolled and randomized into one of the two dietary interventions: a high fiber diet or the American Heart Association (AHA) diet. In the high fiber diet condition, participants received instruction to achieve a daily dietary fiber intake goal of 30 g or more from a variety of food sources. Patients in AHA diet group were instructed to make dietary changes addressing both macro- and micronutrients as recommended by the AHA 2006 dietary guidelines [15].

### 2.2. Participant Characteristics

Demographics including age, race/ethnicity, education, marital status, household income, and employment status were collected at baseline. Weight, height, waist circumference, blood pressure, high-density lipoprotein cholesterol, triglycerides, fasting plasma insulin and glucose, and physical activity were measured at baseline, 6-month, and 12-month visits. Body Mass Index (BMI) was calculated for each time point [BMI = weight (kg)/(height(m))^2^] and was categorized as mild [class I, BMI 30 to 34.99] or moderate (class II, BMI 35 to 40) obesity. Serum was collected after a 12-h fast between the hours of 7:00 am and 10:00 am and stored at −80 °C. To measure insulin, IMMUNLITE 2000 insulin was used, it is a solid-phase, enzyme-labeled chemiluminescent immunometric assay. The SYNCHRON LX System was used to measure glucose, it determines GLUCm concentration by an oxygen rate method employing a Beckman Coulter oxygen electrode. Insulin resistance, assessed by the homeostasis model assessment (HOMA-IR), was calculated as follows: HOMA-IR = fasting plasma insulin (μU/mL) × fasting plasma glucose (mg/dL)/405 [16]. A cutoff of HOMA-IR >3.6 was considered to be insulin resistant [17].

### 2.3. Dietary Assessment

Food intake was assessed at baseline, 6-, and 12-month visits by trained registered dietitians via three 24-h dietary recalls using the Nutritional Data System for Research (NDS-R) software [18], which is developed and annually updated by the Nutrition Coordinating Center, University of Minnesota, Minneapolis, MN. The 24-h dietary recalls were randomly collected on two weekdays and one weekend day by study dietitians within a three-week window at baseline, 6-, and 12-month visits. All participants received a food portion visual booklet prior to receiving the assessment calls to facilitate portion size estimation. The nutrient data collected from 24-h dietary recalls were analyzed.

### 2.4. Statistical Analysis

Since there were no statistically significant differences between the AHA and High Fiber diet group for magnesium intake, we pooled data from both groups to increase statistical power for this secondary analysis. Natural logarithm transformation of HOMA-IR, fasting plasma glucose, and fasting plasma insulin were used since distributions were right-skewed. The outcome variable HOMA-IR was analyzed as both a continuous and a dichotomous variable.

Magnesium intake was included as both a continuous and two types of categorical variables (quartiles or met recommendation). Cut-points for meeting the RDA for magnesium were determined according to the Institute of Medicine of the National Academy of Sciences recommendation for adults: 19–30 years, 400 mg/day (men) and 310 mg/day (women); 31 years and older, 420 mg/day (men) and 320 mg/day (women). This analysis focused on the RDA, not to be confused with the Estimated Average Requirements (EAR), which is actually lower than the RDA. EAR for magnesium for men, ages 19–30, is 330 mg/day and over 31 years and older is 350 mg/day; while for women, ages 19–30, it is 255 mg/day and for 31 and over it is 265 mg/day [19].

Dietary calcium, potassium, sodium, zinc, total calories, total fiber, and ratio of calcium-to-magnesium were considered in bivariate analyses to assess potential confounding. Gender, race/ethnicity, high triglycerides, BMI, and intervention group were considered as covariates due to their potential associations with insulin resistance.

For the continuous metabolic biomarker variables, we used two linear mixed models with random intercepts. In Model 1, we examined the association between dietary magnesium and HOMA-IR and intervention conditions over the 12-month study period; and in Model 2, we examined the association between dietary magnesium and metabolic biomarkers adjusting for important covariates over the study period.

To evaluate the association between magnesium and insulin resistance defined as HOMA-IR more than 3.6, generalized linear mixed models (GLIMMIX) were used to compute the odds ratios (ORs) and 95% CIs. All analyses were performed using SAS version 9.2 software [20].

## 3. Results

At baseline, 6, and 12 months, the dietary magnesium intake was 287 ± 93 mg/day, 307 ± 103 mg/day, and 295 ± 107 mg/day, (*P* = 0.016) and 23.5%, 30.4%, and 27.7% of subjects met the RDA of dietary magnesium intake (χ^2^ = 2.601, *P* = 0.272) (data not shown). Of the subjects, 75% were women and 86% were White. The mean waist circumference, systolic blood pressure, high-density lipoprotein cholesterol (HDL-C), and triglycerides were all higher than the diagnostic standard of MetS, while the mean for diastolic blood pressure and fasting plasma glucose was lower than the diagnostic standards of MetS. Characteristics of subjects at baseline by quartiles of magnesium intake are presented in Table 1. In the highest quartile, there were fewer females, more White individuals and higher total energy and fiber.

**Table 1:** Baseline characteristics according to total magnesium intake quartiles among non-diabetic individuals with metabolic syndrome (*n* = 234).

Characteristics	Dietary Magnesium (mg/day)				*P* Value ^1^
	Q1 (Lowest Quartile, Median = 206.5)	Q2 (Median = 258.3)	Q3 (Median = 322.5)	Q4 (Highest Quartile, Median = 385.2)	
*N*	59	58	58	59	
**Continuous variable ^2^**					
Age (year)	52 ± 11	52 ± 11	52 ± 9	53 ± 10	0.906
Waist circumference (cm)	102 ± 8	101 ± 9	102 ± 9	103 ± 9	0.628
Systolic blood pressure (mmHg)	135 ± 9	138 ± 8	134 ± 12	137 ± 9	0.255
Diastolic blood pressure (mmHg)	80 ± 10	81 ± 9	79 ± 8	81 ± 7	0.724
HDL-C (mg/dL) ^3^	48 ± 10	47 ± 9	48 ± 11	48 ± 11	0.787
Triglycerides (mg/dL)^ 3^	124 ± 62	154 ± 62	168 ± 99	157 ± 72	0.004
Fasting plasma glucose (mg/dL)^ 3^	100 ± 15	100 ± 12	96 ± 12	100 ± 11	0.347
Fasting plasma insulin (μU/mL)^ 3^	17 ± 12	13 ± 12	16 ± 16	12 ± 9	0.068
HOMA insulin resistance ^3^	4.2 ± 3.4	3.4 ± 3.7	3.9 ± 4.1	3.0 ± 2.5	0.107
Total fiber intake (mg/day)	13 ± 6	16 ± 3	21 ± 4	26 ± 6	<0.001
Total energy (kcal/day)	1392 ± 405	1691 ± 393	1974 ± 453	2452 ± 732	<0.001
Physical activity (MET: h/day)	27 ± 3	28 ± 4	28 ± 4	29 ± 5	0.117
**Categorical variable [*n* (%)]**					
**Female**	51 (86%)	40 (69%)	44 (76%)	35 (59%)	0.009
**White**	44 (75%)	54 (93%)	53(91%)	57 (97%)	<0.001
**Married**	35 (59%)	47 (81%)	36 (62%)	38 (64%)	0.057
**Education**					
High school or less	10 (17%)	6 (10%)	9 (15%)	7 (12%)	0.709
Bachelor’s degree or less	36 (61%)	36 (62%)	38 (66%)	32 (56%)	
Graduate/professional	13 (22%)	16 (28%)	11 (19%)	18 (32%)	
**Household income**					
$0–$30,000	8 (14%)	3 (5%)	8 (14%)	5 (8%)	0.431
$30,000–$50,000	8 (14%)	13 (22%)	9 (16%)	11 (19%)	
$50,000–$75,000	13 (22%)	7 (12%)	9 (16%)	9 (16%)	
More than $75,000	13 (22%)	25 (43%)	20 (34%)	20 (34%)	
Unclear	17 (29%)	10 (17%)	12 (21%)	14 (24%)	
**Employed full-time or part-time**	18 (31%)	11 (19%)	12 (21%)	9 (16%)	0.243
**BMI (30–34.9 kg/m^2^)**	21 (36%)	33 (57%)	32 (55%)	27 (46%)	0.046

HDL-C: High-density lipoprotein cholesterol; HOMA: Homeostasis model of assessment; BMI: Body mass index; ^1^ *P* values are for any difference across the quintiles of magnesium intake using ANOVA, or χ^2^ test as appropriate; ^2^ All continuous variables values are mean ± standard deviation; ^3^ *P* values based on log-transformed outcomes. Values presented in rows have been transformed back to original units.

Table 2 presents the regression coefficients (β) of dietary magnesium intake and metabolic biomarkers of insulin resistance using a linear mixed model. Time-point and group were analyzed in Model 1; Model 2 included additional adjustment for gender, race/ethnicity, triglycerides, and BMI category. For both Model 1 and Model 2 significant inverse associations were observed between metabolic biomarkers of insulin resistance and magnesium intake. High BMI and female participants showed significant positive association with metabolic biomarkers and high insulin resistance status in linear mixed models. As total calories, total fiber, sodium, potassium, calcium, zinc, and ratio of calcium-to-magnesium could be potential confounders, we also evaluated their relationship with metabolic biomarkers using the same statistical models (data not shown). The only significant relationship with metabolic biomarkers was magnesium intake.

**Table 2:** Regression coefficients (β) of magnesium intake and metabolic biomarkers of insulin resistance according to a linear mixed model among non-diabetic individuals with metabolic syndrome (*n* = 234) ^1^.

Dietary factors	Model 1 ^2^			Model 2 ^3^		
	β	95% CI	*P*	β	95% CI	*P*
Ln insulin	−0.00073	−0.00133, −0.00013	0.016	−0.00095	−0.00155, −0.00035	0.002
Ln glucose	−0.00009	−0.00019, −0.00001	0.048	−0.00015	−0.00024, −0.00005	0.002
Ln HOMA-IR	−0.00082	−0.00145, −0.00019	0.011	−0.00110	−0.00173, −0.00046	<0.001

CI: confidence intervals; HOMA-IR: Homeostasis model of assessment-Insulin resistance; Ln: natural logarithm; ^1^ Estimated by PROC MIXED in SAS; ^2^ Adjusted for group and time-point; ^3^ Adjusted for gender, race/ethnicity, BMI category, triglycerides, group, and time-point.

Total energy and total dietary fiber were highly correlated with magnesium intake (*r* = 0.62 and *r* = 0.78, respectively; both *P* values < 0.001). We analyzed Model 2 with additional adjustment for calories and fiber and results were slightly attenuated toward the null, however significant associations for magnesium remained (data not shown).

To examine the magnesium dose-response, and if the RDA for intake was met, we examined quartiles of magnesium intake with the outcome of HOMA-IR >3.6 (Table 3). Magnesium intake category variables were assessed over three time-points using linear mixed models. After adjustment for covariates, the likelihood of elevated HOMA-IR (>3.6) over time was 71% lower (OR: 0.29; 95% CI: 0.12, 0.72) in participants in the highest quartile of dietary magnesium intake than those in the lowest quartile at baseline. The multivariate-adjusted OR of high HOMA-IR over time for those that did not meet the magnesium RDA was 0.37 (95% CI: 0.18, 0.77). Evaluation of the association between HOMA-IR over time by magnesium intake at baseline, indicated similar odds ratios [(0.19, 95% CI: 0.06, 0.59) and (0.33, 95% CI: 0.13, 0.85), respectively] (data not shown in Table 3).

**Table 3:** Odds ratios (OR) and 95% conference intervals (CIs) of elevated HOMA-IR (>3.6) over three time-points from a linear mixed model among non-diabetic individuals with metabolic syndrome (*n* = 234) ^1^.

Dietary magnesium intake (mg/day)	Unadjusted		Adjusted ^2^	
	OR	95% CI	OR	95% CI
Quartile 1 **(Median = 206.5)**	1.00	Referent	1.00	Referent
Quartile 2 **(Median = 258.3)**	0.51	0.23, 1.11	0.49	0.22, 1.08
Quartile 3 **(Median = 322.5)**	0.82	0.36, 1.87	0.79	0.35, 1.79
Quartile 4 **(Median = 385.2)**	0.40	0.16, 0.98	0.29	0.12, 0.72
Not met RDA	1.00	Referent	1.00	Referent
Met RDA	0.42	0.20, 0.88	0.37	0.18, 0.77

OR: odds ratio; CI: confidence intervals; HOMA-IR: Homeostasis model of assessment-Insulin resistance; RDA: recommended Daily Allowance; ^1^ Estimated by PROC GLIMMIX in SAS; ^2^ Adjusted for gender, marital status, race/ethnicity, BMI category, triglycerides, group, and time-point.

## 4. Discussion

In this study, we found an inverse association between magnesium intake and insulin, glucose, and HOMA-IR, and that a higher level of magnesium intake from foods was protective against insulin resistance in non-diabetic participants with MetS. Magnesium is required for glucose utilization and insulin signaling. Lower intake of foods rich in magnesium may lead to metabolic alterations in cellular magnesium and may affect the development of insulin resistance by altering the glucose entry into the cell [5,21]. Some dietary factors may confound the relationship between magnesium intake and insulin resistance, as magnesium occurs in foods that also contain other food components, such as phytonutrients, fiber, and other minerals. However, in our study, metabolic biomarkers of insulin resistance were tested for associations with these potential confounders using the same model with magnesium intake, and were found to be non-significant, suggesting that dietary magnesium may improve insulin resistance independently. Our findings are consistent with results from previous studies that cite the role of magnesium as a crucial component in improving insulin resistance [22,23] Prior studies examining magnesium and insulin resistance confirm our findings, though more data is needed to continue further study for patients with metabolic syndrome. Although our study examined RDA, according to a publication by Hunt and Johnson, most Americans do not meet the EAR for magnesium, and since data on EAR is derived from a small amount of studies, they suggest that a reduction in the magnesium requirement for healthy men and women is warranted [24]. If the standards are further reduced in the future, it would be interesting to examine the impact these recommendations might have in a population with metabolic syndrome as our study found only a small percentage of participants who were able to meet the magnesium RDA. Indeed, further inquiry into the appropriate threshold of magnesium intake is needed to address population health in the United States.

Our study has several limitations. Our intervention focused only on food derived items and not on supplements, but would make for an interesting topic of further study as Fulgoni *et al*. noted that there is “very limited information on usual micronutrient intakes from dietary supplements in the United States population (with the exception of folate)” [25]. The possibility of confounding from supplements or unknown factors cannot be ruled out, though the potential misclassification will result in attenuation of inverse association. In addition this study measured magnesium intake using three 24-h dietary recalls, which have the potential to be subject to reporting bias, as study participants with MetS have been shown to systematically underreport their food intake [26]. Despite these potential drawbacks, 24-h dietary recalls are currently considered the “gold standard” of dietary research for micronutrients [27].

This investigation also has several strengths, including its longitudinal study design using non-diabetic participants with MetS. The majority of previous findings are cross-sectional studies using normal populations or those with diabetes. Although previous studies have evaluated the protective effect of magnesium, satisfying the RDA is a simple message that can have important clinical meaning. Our findings offer a unique contribution that meeting the RDA for magnesium may demonstrate a protective effect on insulin resistance.

## 5. Conclusions

This study observed a low percentage of participants who actually met the magnesium RDA, suggesting that increasing dietary magnesium to meet the RDA is associated with improving insulin resistance among non-diabetic individuals with MetS. Since this population has a higher risk of cardiovascular disease and type 2 diabetes, dietary behaviors that have the ability to impact insulin resistance can have far-reaching clinical implications. The results of this study merit further investigation into the understudied role of adequate magnesium intake and its role in improving insulin resistance in this population.
